# Kinome Profiling to Predict Sensitivity to MAPK Inhibition in Melanoma and to Provide New Insights into Intrinsic and Acquired Mechanism of Resistance Short Title: Sensitivity Prediction to MAPK Inhibitors in Melanoma

**DOI:** 10.3390/cancers12020512

**Published:** 2020-02-22

**Authors:** Mohamad Krayem, Philippe Aftimos, Ahmad Najem, Tim van den Hooven, Adriënne van den Berg, Liesbeth Hovestad-Bijl, Rik de Wijn, Riet Hilhorst, Rob Ruijtenbeek, Malak Sabbah, Joseph Kerger, Ahmad Awada, Fabrice Journe, Ghanem E. Ghanem

**Affiliations:** 1 Laboratory of Oncology and Experimental Surgery, Institut Jules Bordet, Université Libre de Bruxelles, 1000 Brussels, Belgium; ahmad.najem@bordet.be (A.N.); msabbah@ulb.ac.be (M.S.); ahmad.awada@bordet.be (A.A.); fabrice.journe@bordet.be (F.J.); gghanem@ulb.ac.be (G.E.G.); 2 Medical Oncology Clinic, Institut Jules Bordet, Université Libre de Bruxelles, 1000 Brussels, Belgium; philippe.aftimos@bordet.be (P.A.); joseph.kerger@bordet.be (J.K.); 3 PamGene International BV, 5211HH ’s-Hertogenbosch, The Netherlands; timvdhooven@gmail.com (T.v.d.H.); avdberg@pamgene.com (A.v.d.B.); lhovestad@pamgene.com (L.H.-B.); rdwijn@pamgene.com (R.d.W.); rhilhorst@pamgene.com (R.H.); rru@genmab.com (R.R.)

**Keywords:** MAPK pathway, phosphotyrosine kinase, serine-threonine kinase, kinase inhibitors, intrinsic and acquired resistance

## Abstract

Mitogen-activated protein kinase (MAPK) inhibition with the combination of BRAF (Rapidly Accelerated Fibrosarcoma) and MEK (Mitogen-activated protein kinase kinase) inhibitors has become the standard of first-line therapy of metastatic melanoma harbouring BRAF V600 mutations. However, about half of the patients present with primary resistance while the remaining develop secondary resistance under prolonged treatment. Thus, there is a need for predictive biomarkers for sensitivity and/or resistance to further refine the patient population likely to benefit from MAPK inhibitors. In this study, we explored a top-down approach using a multiplex kinase assay, first, to discover a kinome signature predicting sensitivity, intrinsic and acquired resistance to MAPK inhibitors in melanoma, and second, to understand the mechanism of resistance using cell lines. Pre-dose tissues from patients (four responders and three non-responders to BRAFi monotherapy) were profiled for phosphotyrosine kinase (PTK) and serine-threonine kinase (STK) activities on a PamChip^®^ peptide microarray in the presence and absence of ex vivo BRAFi. In addition, molecular studies were conducted on four sensitive parental lines, their offspring with acquired resistance to BRAFi and two lines with intrinsic resistance. PTK and STK activities in cell lysates were measured in the presence and absence of ex vivo BRAFi and/or MEKi. In tissue lysates, concentration-dependent *ex vivo* inhibition of STK and PTK activities with dabrafenib was stronger in responders than in non-responders. This difference was confirmed in cell lines comparing sensitive and resistant ones. Interestingly, common features of resistance were increased activity of receptor tyrosine kinases, Proto-oncogene tyrosine-protein kinase Src (Src) family kinases and protein kinase B (PKB, AKT) signalling. These latter results were confirmed by Western blots. While dabrafenib alone showed an inhibition of STK and PTK activities in both tissues and cell lines, the combination of dabrafenib and trametinib showed an antagonism on the STK activities and a synergism on PTK activities, resulting in stronger inhibitions of overall tyrosine kinase activities. Altogether; these data reveal that resistance of tumours and cell lines to MAPK inhibitors can be predicted using a multiplex kinase assay and is associated with an increase in specific tyrosine kinase activities and globally to AKT signalling in the patient’s tissue. Thus, such a predictive kinome signature would help to identify patients with innate resistance to MAPK double inhibition in order to propose other therapies.

## 1. Introduction

Melanoma is a malignant tumour that arises from the malignant transformation of melanocytes. It particularly affects young adults as it is the third most frequent cancer in the 20–39 year old age range. It is the least common but the most aggressive skin cancer, accounting for the vast majority of skin cancer deaths [1]. Historically, the survival rate at 10 years for patients with metastatic melanoma has been less than 10% [1]. The incidence of melanoma of the skin has risen rapidly over the past 30 years and its associated mortality has strongly increased [2,3].

The identification of the signalling pathways that are central to melanoma initiation and progression inaugurated a new era of treatments of both metastatic and early stage melanoma, with novel agents targeting specific genomic alterations underlying melanoma initiation and progression [4]. UltraViolet-B radiation causes upregulation of cyclic AMP (cAMP) signalling in response to α-melanocyte stimulating hormone binding to specific melanocyte receptors. This cAMP-dependent cascade activates the mitogen-activated protein kinase (MAPK) pathway [5], consisting of the RAS guanosine triphosphate hydrolase (GTPase) and the RAF, MEK, and Extracellular signal-regulated kinase (ERK kinases), and is activated in up to 90% of all melanomas [6]. BRAF, a serine/threonine specific protein kinase, is mutated in 50% to 70% of cutaneous melanomas. The most common mutation in BRAF, in 80% of cases, is a glutamic acid for valine substitution at position 600 (V600E) [7]. ^V600E^BRAF is activated almost 500 fold [8], it transforms immortalised melanocytes [9], and it stimulates proliferation and survival in melanoma cells [10]. ^V600E^BRAF also stimulates melanoma cell invasion in vitro and is important for tumour neo-angiogenesis in vivo. 

In this context, BRAF inhibitors (BRAFi) have generated remarkable clinical responses in a high proportion of melanoma patients harbouring ^V600^BRAF mutations [11,12,13]. Indeed, early studies with vemurafenib monotherapy showed an impressive objective response rate, albeit short-lasting [11]. These results were replicated with other BRAF inhibitors such as dabrafenib [14] and with MEK inhibitors (MEKi) such as trametinib [15]. Combination therapy with a BRAFi and a MEKi proved superior to monotherapy and is currently the standard of care treatment with three approved combinations: dabrafenib + trametinib [16], vemurafenib + cobimetinib [17], encorafenib + binimetinib [18]. The combination of dabrafenib + trametinib also received approval in the adjuvant treatment of lymph- node-positive melanoma with a BRAF V600 mutation [19]. However, despite the rapid response and short-term increases in patient survival, primary/intrinsic resistance and secondary/acquired resistance frequently developed [20,21,22]. 

Targeting specific oncoproteins crucial for tumour growth, such as BRAF, induces adaptive kinome responses that upregulate alternative kinase signalling to overcome the inhibitor treatment [20,22,23]. This kinome reprogramming is mechanistically based on the disruption of feedback and feedforward regulatory loops that serve to bypass the inhibition and rapidly elicit resistance to targeted therapies [24]. Adaptive responses can be triggered that counteract the initial dependence of tumour cells; this rerouting of signalling pathways is a major reason why kinase inhibitors often do not show durable responses in the treatment of cancer patients. 

Thus, biomarkers of resistance mechanisms are searched to identify patients in need of novel drug combinations. In this study, we explored a top-down approach using an innovative multiplex kinase assay to identify kinome profiles of individuals not responding to therapy using baseline biopsy samples, and to further understand the mechanism of resistance using patient-derived cell lines characterised for sensitivity, intrinsic and acquired resistance to the MAPK inhibitors.

## 2. Materials and Methods

### 2.1. Reagents

Vemurafenib (PLX4032) was from Nuclilab (Ede, The Netherlands), dabrafenib and trametinib were from Selleck Chemicals (Houston, TX, USA). All were dissolved according to the manufacturer’s recommendation in DMSO at 10^−2^ M, aliquoted and stored at −20 °C.

### 2.2. Melanoma Cell Lines

^V600E^BRAF mutant human melanoma cell lines (MM043, MM050, MM054, MM164 and MM074) (Table S1) used in this study were all established in the Laboratory of Oncology and Experimental Surgery [23,25] and SKMEL-28 is an ATCC^®^ HTB72™ melanoma cell line [21]. The BRAF mutation was evaluated using a targeted gene-sequencing panel of 48 genes (TruSeq Amplicon-Cancer Panel, Illumina, San Diego, CA, USA). Cells harbouring the BRAF V600E mutation with acquired resistance to vemurafenib were obtained after chronic treatment with increasing concentrations (0.1–2 μM) of the drug for 12 weeks (Figure S2A) [21,26].

### 2.3. Cell Culture Conditions

Cells were grown in HAM-F10 medium supplemented with 5% heat-inactivated foetal calf serum, 5% heat-inactivated newborn calf serum and with L-glutamine, penicillin and streptomycin at standard concentrations (all from Thermo Fischer Scientific, Waltham, MA, USA) (culture medium) at 37 °C in a humidified 95% air and 5% CO_2_ atmosphere. For routine maintenance, cells were propagated in flasks, harvested by trypsinization (0.05% trypsin-EDTA) (Thermo Fischer Scientific, Waltham, MA, USA) and sub-cultured twice weekly. Cell count and volume were evaluated using a TC10™ Automated Cell Counter (Bio-Rad, Hercules, CA, USA). All cell lines were regularly checked for mycoplasma contamination using MycoAlert^®^ Mycoplasma Detection Kit (Lonza, Rockland, ME, USA).

### 2.4. Patients and Tissue Collection

This retrospective study was approved by the Central Ethics Committee of the Institut Jules Bordet (CE2023). All patients included in this study received information regarding the use of residual human corporal materials and clinical data for researches and signed an informed consent form prior to biopsy. Snap-frozen in liquid nitrogen biopsies (all from skin lesions or lymph nodes) were collected from seven patients with metastatic melanoma harbouring the BRAF V600E mutation prior to the initiation of first-line therapy with vemurafenib. Biopsies were stored at −80 °C until use. Four patients were responders and three patients were non-responders to the therapy. Responders were patients who had achieved a partial or complete response as best response using Response Evaluation Criteria In Solid Tumors (RECIST) 1.1 [27] or PET Response Criteria in Solid Tumors (PERCIST) [28]. Duration of response varied from five months to still ongoing at five years. All non-responders had progressive disease as best response. This study was performed in accordance with the REMARK guidelines [29,30].

### 2.5. Lysis of Tissues and Cells

Frozen tissues were Tek^®^ OCTTM embedded and cut in a microtome at −20 °C. Tissue sections of 5 µm were cut for histological evaluation (HE staining to assess the percentage of tumour) while 60 µm tissue sections (three sections in separate vials) were cut for protein extraction. Resistant cell lines were cultured in the presence of 2 µM vemurafenib. Before lysis of cell lines, culture medium with or without 2 µM vemurafenib was removed from the cells and the cells were washed with ice-cold PBS prior to protein extraction.

Tissues and cells were lysed on ice with M-PER (Mammalian Protein Extraction Reagent, Thermo Fischer Scientific, Waltham, MA, USA) lysis buffer containing 1/100 Protease Inhibitor Cocktail EDTA-free (Thermo Fischer Scientific) and 1/100 Phosphatase Inhibitor Cocktail (Thermo Fischer Scientific). After centrifugation (10 min, 4 °C, 10,000× g), the supernatants were aliquoted, snap-frozen in liquid nitrogen and stored at −80 °C. The protein concentration was determined using the BCA Assay (Thermo Fischer Scientific, Waltham, MA, USA) with bovine serum albumin (BSA) as the standard. For each experiment, a never thawed aliquot was used.

### 2.6. Kinase Activity Profiling on PamChip^®^ Peptide Microarrays 

Kinase activity profiles were determined using the PamChip^®^ 96 or PamChip^®^ 12 serine/threonine (STK) and protein tyrosine (PTK) peptide microarray system from PamGene International B.V. (’s-Hertogenbosch, The Netherlands) according to the instructions of the manufacturer, essentially as described in Rosenberger et al. [31]. Of note, arrays were blocked with 2% BSA instead of Odyssey blocking buffer, and the assay buffer contained EDTA instead of EGTA. For STK, tissue lysates (1 µg protein/array) were incubated with 0, 1, 10 or 25 μM dabrafenib, while for PTK, 5 µg protein/array was exposed to 0, 0.1, 0.2 or 0.5 µM dabrafenib. Sample input for cell line lysates was 1 µg/array in STK assays and 5 µg/array for PTK assays. Lysates were tested with 10 µM (STK) or 0.5 µM (PTK) dabrafenib, 10 µM trametinib, or 10 µM selumetinib. The ATP concentration was 100 µM in a total assay volume of 40 µL. All incubations, including the controls, contained 2% DMSO. Experiments were performed in triplicate. Images of the arrays were captured over time and after 60 min of incubation, after a washing step.

### 2.7. Data Analysis and Quality Control of the PamChip^®^ Peptide Microarrays

Data quantification of the array images at all exposure times and time points and visualization of the data were performed using BioNavigator software (PamGene International B.V., ’s-Hertogenbosch, The Netherlands). BioNavigator automatically grids images taken by the PamStation and quantifies the signal and background of each peptide spot, providing a signal minus background value at multiple times throughout the incubation at multiple exposure times. These values were combined into one value for each time point. For the STK assay, peptides having a signal above the background in more than 80% of the arrays, i.e., 126/144 peptides were retained for the analysis. For the PTK assay, only peptides showing an increase of signal in time, essentially identifying phosphorylation kinetics in more than 80% of the arrays, i.e., 90/142 peptides, were retained for analysis.

Values were 2log-transformed for visualization. To identify peptides that are significantly different between different conditions, statistical tests were performed, such as Student’s t-test, Paired t-test, one-way ANOVA or a mixed model analysis.

For upstream kinase analysis, kinases known to phosphorylate the phosphorylation sites in the peptides were identified in databases. Based on phosphorylation patterns and permutation tests, the kinases most likely to cause the effects were identified, using Bionavigator sofware [32]. STRING (https://string-db.org/) was used to create functional protein interaction networks. Kinome phylogenetic trees were made in Kinmap (http://www.kinhub.org/kinmap/).

### 2.8. Western Blot Analysis

Cells were plated in Petri dishes (3 × 10^6^ cells/dish) in culture medium. One day after plating, the culture medium was replaced by fresh medium and cells were grown for two more days. Then, cells were exposed to effectors or vehicle for 24 hours. Cells were lysed using a detergent cocktail and extracted proteins were analysed by Western blotting as previously described [23,25]. Equal amounts of extracted proteins (35 µg) were subjected to 10% or 12% SDS-PAGE and electrotransferred onto nitrocellulose membranes using an iBlot^®^ Dry Blotting System (Invitrogen, Life Technologies, Gent, Belgium). Immunodetections were performed using antibodies raised against pAKT (Ser 473) (D9E, 1/500), AKT (40D4, 1/1000), pSrc (Tyr416) (D49G4, 1/1000), Src (2108, 1/1000), Hepatocyte growth factor receptor MET (D1C2, 1/1000), Epidermal Growth Factor Receptor (EGFR) (D6B6, 1/1000) (all from Cell Signaling Technology, Danvers, MA, USA), pERK (Tyr 204) (E-4, 1/1000), ERK2 (C-14, 1/2000) (from Santa Cruz Biotechnology, Santa Cruz, CA, USA), and β-actin (C4, 1/5000) (from Millipore, Temecula, CA, USA).

Peroxidase-labelled anti-rabbit IgG antibody (1/5000) or peroxidase-labelled anti-mouse IgG antibody (1/5000) (both from Amersham Pharmacia Biotech, Roosendaal, The Netherlands) were used as secondary reagents to detect corresponding primary antibodies. Bound peroxidase activity was revealed using the SuperSignal^®^ West Pico Chemiluminescent Substrate (Thermo Fischer Scientific, Waltham, MA, USA) following the manufacturer’s indications. Immunostaining signals were digitalised with a PC-driven LAS-3000 CCD camera (Fujifilm, Tokyo, Japan), using a software specifically designed for image acquisition (Image Reader, Raytest^®^, Straubenhardt, Germany). Immunoreactive band intensities were quantified using the software AIDA^®^ Image Analyser 3.45 (Raytest^®^).

## 3. Results

### 3.1. Kinome Profiling Reveals Differential Activity between Responder and Non-Responder Patients to BRAF Inhibition

**Figure 1:**
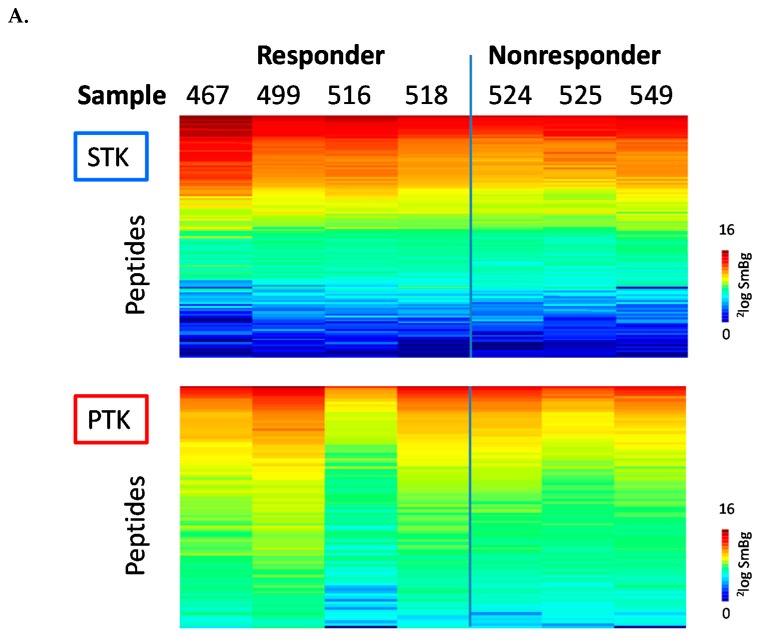

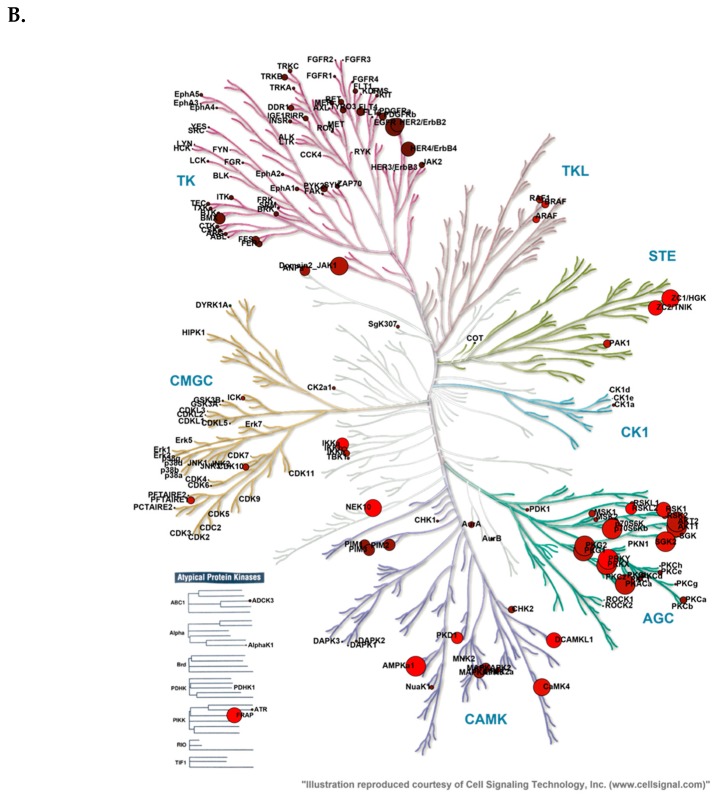
Serine/threonine and tyrosine kinase activities of seven pre-treatment melanoma biopsies harbouring the BRAF V600E mutation. (**A**) Serine Threonine kinase (STK, top) and tyrosine kinase (PTK, bottom) activity profiles in clinical responders and non-responders to vemurafenib. Log_2_ transformed signals represented as a heat map. Rows represent peptides, sorted on overall mean signal intensity. Columns represent the samples; (**B**) Upstream kinase analysis of basal kinase activity profiles of pre-treatment melanoma tumour samples to identify kinases that show higher activity in the non-responder groups compared to the responders. A brighter colour corresponds with more significant differences. Illustration reproduced courtesy of Cell Signaling Technology, Inc. (www.cellsignal.com) using the Kinmap.

**Figure 2:**
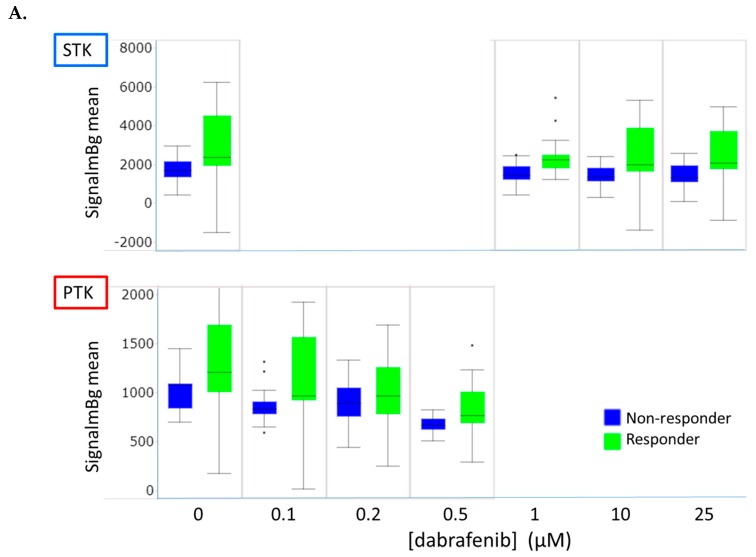

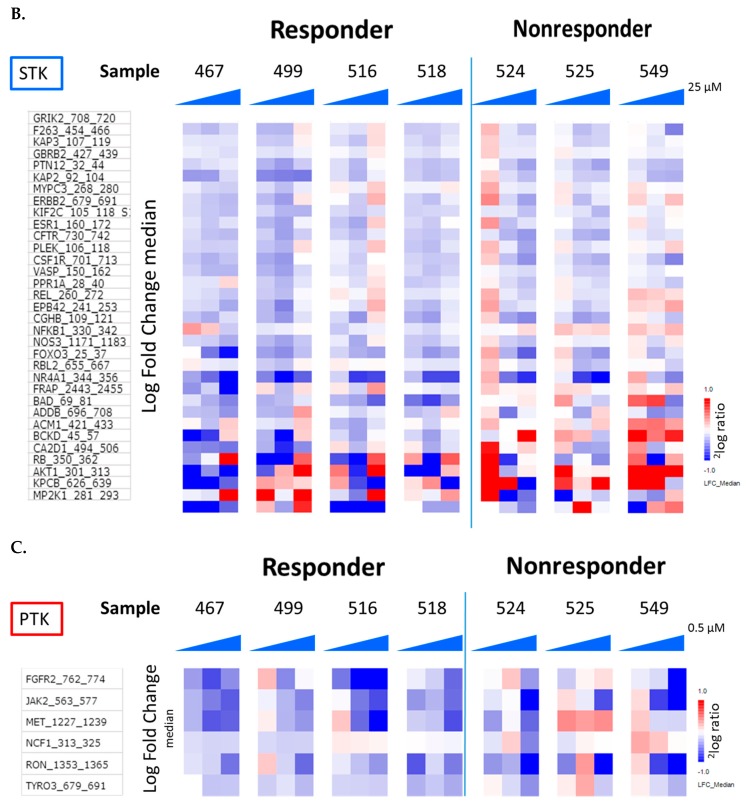

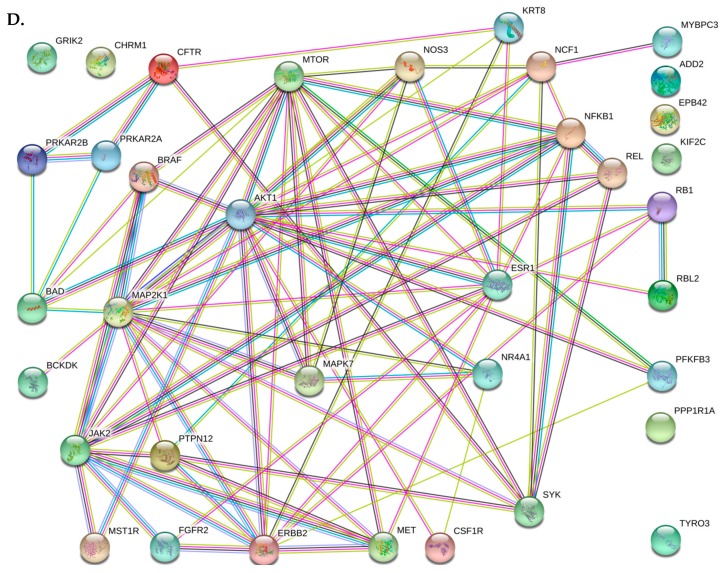
Effect of dabrafenib on kinase activity of the melanoma tissues. (**A**) Clinical responders and non-responders to vemurafenib: Median signal intensity of all peptides for serine/threonine and tyrosine kinase activity as a function of dabrafenib concentration; (**B**,**C**) Log_2_ Fold Change of kinase activity with dabrafenib concentration. Only peptides that show significant (*p* < 0.05) difference in inhibition between responder and non-responders are shown. For STK (**B**), lysates were incubated with 0, 1, 10 or 25 µM dabrafenib (*n* = 3). For PTK (**C**), lysates were incubated with 0, 0.1, 0.2 or 0.5 µM dabrafenib (*n* = 3); (**D**) Network of parental protein for peptides that differentiated clinical responders from non-responders based on inhibition by dabrafenib. Network was made in STRING, using peptides with *p* < 0.05 for STK with 1 and 10 µM and for PTK with 0.1, 0.2 and 0.5 µM dabrafenib. The BRAF identity was added to illustrate its position in the network.

We performed first a kinome profiling of melanoma samples from patient tissues using a phosphorylation-based kinome array (PamChip^®^) that assesses both serine/threonine kinase (STK) and protein tyrosine kinase (PTK) activities. The experimental workflow is represented in Figure S1. Among the seven patients, four patients were responders\sensitive (no. 467, 499, 516 and 518) while three patients were non-responders\intrinsic resistant (no. 524, 525, and 549) (Table S2). The level of phosphorylation per peptide for each sample was determined, and the data were visualised in heat maps for STK and PTK activity (Figure 1A). The variation between the samples was large, but responders tended to have higher overall phosphorylation levels than non-responders in both the STK and the PTK assays (Figure 2A, 0 µM dabrafenib). Statistical analysis found only four peptides (one for PTK and three for STK) that had significantly different signal intensities (*p* < 0.05) between responders and non-responders. For PTK, a peptide derived from the Ephrin Receptor B4 (EphB4) had *p* < 0.05 between the two groups. For STK, one peptide (derived from FKBP12-rapamycin complex-associated protein, (FRAP), aka Mammalian target of rapamycin (mTOR)) contains the AKT phosphorylation site of mTOR, the other is a Protein Kinase C (PKC)-derived peptide and the third is derived from the Neurotrophic tyrosine kinase receptor type 3 (NTRK3) (involved in several cancers, e.g., melanoma) [33].

The peptides present on the array are phosphorylated by kinases in the tissue lysates. To generate a hypothesis about kinases in the lysate responsible for this phosphorylation, without a priori assumptions, we performed an upstream kinase analysis. Based on the relatively small differences between the responders and non-responders, the upstream kinase analysis suggested an increased activity in responders of AKT, p70S6K (p70 ribosomal S6 kinase), mTOR, PKA (Protein Kinase A) and/or PKC (Protein Kinase C) and members of the EGFR family (Figure 1B). Thus, the basal kinase activity profiles of melanoma tumour tissue showed a wide variation in kinase activity, making it difficult to identify robust common effects that could distinguish responders from non-responders.

### 3.2. Effect of a ^V600E^BRAF Inhibitor on Kinase Activity in Melanoma Tissue Lysates

We evaluated the inhibition profiles obtained with ex vivo exposure of melanoma tumour lysates to a ^V600E^BRAF inhibitor (dabrafenib) with two purposes: (1) to gain a more comprehensive insight into the pathways that have an impact on the response to BRAF inhibitors, and (2) to distinguish responders from non-responders based on relative signal intensities (log fold change inhibited/non-inhibited) to overcome the differences in basal kinase activity between the patients.

Dabrafenib is an inhibitor of ^V600E^BRAF, which is 7 and 9 fold less potent to BRAF WT and CRAF, respectively [34]. The overall effect of dabrafenib on both PTK and STK activity is visualised by calculating for each technical replicate the median signal intensity of all peptides and representing the mean of the technical replicates in box plots. This revealed a stronger inhibition of the kinase activity in responders than in non-responders (Figure 2A). These data could suggest that dabrafenib has a potent off-target effect on tyrosine kinases in line with observations reported by Uitdehaag et al. [35].

Effect of dabrafenib addition to lysates on kinase activity was expressed as Log Fold Change (LFC) vs. the incubation without inhibitor. Statistical analysis of LFCs was used to identify a subset of peptides that showed significantly different inhibition between the responders and the non-responders at any of the inhibitor concentrations used (Figure 2B,C and Table S3). At 1 µM dabrafenib, the responders showed more inhibition of STK activity than the non-responders. This was observed, for example, for a RAF substrate peptide (RBL_655_667) and an AKT substrate peptide (FRAP_2443_2454 (aka mTOR)). At higher dabrafenib concentrations, the effects become more sample specific. Off-target effects at higher inhibitor concentrations could be an explanation. In addition, among the peptides showing significantly higher inhibition of PTK activity in the responders than in the non-responders, we noticed four peptides derived from RTKs (receptor tyrosine kinases) such MET, RON (Recepteur d’Origne Nantais), FGFR (Fibroblast growth factor receptor) and TYRO-3 (Tyrosine-protein kinase SKY). The peptides from STK and PTK analyses that significantly differentiated responders from non-responders (*p* < 0.05) (25 µM inhibitor was excluded to avoid off-target effects) were linked to the UniProt ID of their parental protein and subjected to a network analysis in STRING. Each protein was represented as a node with edged interactions. The analysis shows a strong connection between MEK (aka MP2K1) and AKT (PKB) and mTOR (Figure 2D). Upstream kinase analysis indicates that the peptides that show differential inhibition represent substrates for SRC family kinases, PKA, PKC, AKT and PIM (Proviral insertion in murine) family kinases that are implicated in the regulation of cell growth, cell cycle progression and metabolism. Altogether, these results suggest that responders show more inhibition by dabrafenib on their PTK (SRC family kinases) and STK activity, notably the MEK-AKT-mTOR axis and can thus be differentiated from non-responders.

### 3.3. Comparative Analysis of Kinome Profiles between Melanoma Cell Lines Sensitive and Resistant to a ^V600E^BRAF Inhibitor

To explore the mechanisms of resistance mediated by kinome reprogramming after cell line exposure to BRAFi, we compared the kinome profiles in BRAFi sensitive and resistant cell lines to identify the activated kinases in BRAF-resistant cells from deep kinome data. Four melanoma cell lines (MM050, MM074, MM164 and SKMEL-28), all with BRAF V600E mutations, were made resistant to the BRAF V600E inhibitor vemurafenib after chronic exposure for 12 weeks to gradually increasing concentrations of vemurafenib (0.1–2 µM) (Figure S2A). Acquired resistance to vemurafenib translated into a 15- to 210-fold increase of IC^50^ in resistant cells as compared to parental cell lines (Table S1, Figure S2B,C).

**Figure 3:**
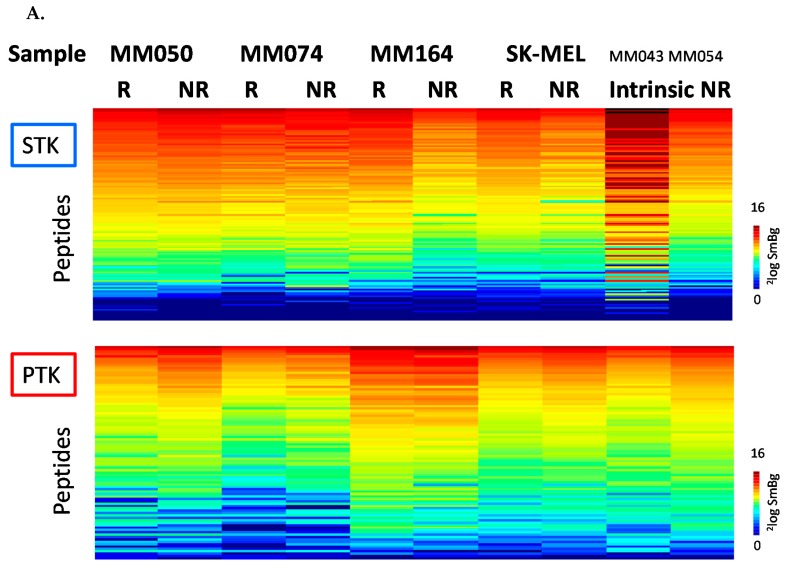

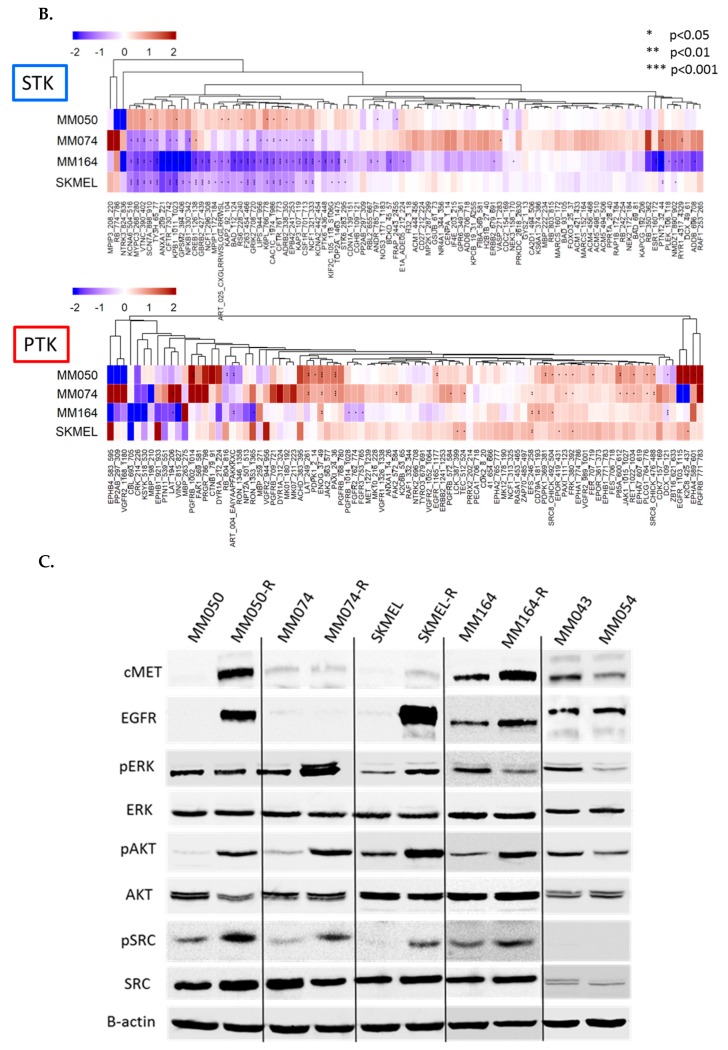
Comparative analyses of kinome profiles between BRAFi sensitive and resistant melanoma cell lines. (**A**) Serine Threonine kinase (STK, top) and tyrosine kinase (PTK, bottom) activity profiles cell lines sensitive (S), with acquired resistance (AR) or intrinsically resistant (IR) to vemurafenib; (**B**) Peptides that show significant (*p* < 0.05) difference in inhibition between cell lines with acquired resistance to vemurafenib: effects per cell line. ^2^Log Fold Change vs. parental cell line. Red—increase, blue—decrease in resistant cell line. Asterisks indicate significance; (**C**) Western Blot analysis of key targeted protein in MAPK, PI3K/AKT and SRC signalling pathways in four BRAFi sensitive, and six cell lines with intrinsic (MM043 and MM054) and acquired resistance to vemurafenib, (R refers to acquired resistant cells compared to parental sensitive cells).

However, the cell lines with intrinsic resistance showed opposing effects. For example, the MM043 cells, an intestinal metastatic cell line with intrinsic resistance to vemurafenib, showed a very different STK activity profile as compared to the other cell lines (Figure 3A). Statistical analysis of the differences between the sensitive cell lines and the two intrinsically resistant cell lines yielded no common effects, as 1 and 0 phosphorylated peptide was reported to be significantly different (*p* < 0.05) in the STK and PTK assays, respectively, suggesting that intrinsic resistance is not related to the activation of specific kinases.

Cells with acquired resistance to vemurafenib (except for MM164) show higher tyrosine kinase activity as evidenced by higher phosphorylation levels of peptide substrates, compared to the sensitive counterpart (Figure 3A). STK activity is higher in MM050-R and MM074-R but lower in SKMEL-R and MM164-R, compared to their parental cell line. Using t-tests, peptides that significantly differ between sensitive and resistant cell line pairs were identified (Figure 3B and Table S3). For the interpretation of the mechanism of acquired resistance an upstream kinase analysis was performed for each pair. Results for each pair were projected on the kinome phylogenetic tree (Figure S3). In all cell lines with acquired resistance a higher activity of SRC family kinases is suggested, as well as more activity of HER (human epidermal growth factor receptor) family kinases in MM050-R and SK-MEL-28-R, but not in MM074-R. Also, the resistant cell lines (excepting the MM050-R) are suggested to have reduced PKA activity compared to the parental cell lines.

Next, we analysed the protein expression of significantly regulated kinases to evaluate whether the comparative analyses of kinome profiles between the sensitive and the resistant cell lines are reflected in altered levels of phosphorylation of target proteins by Western blot (Figure 3C). An upregulation of RTK receptors such EGFR and MET was observed in the resistant cell lines (MM050-R; SKMEL-R and MM164-R), along with an increase in AKT and SRC phosphorylation in all cell lines with acquired resistance. ERK phosphorylation increased in two lines (MM074-R and SKMEL-R), these results confirm the activation of the phosphatidylinositol 3-kinase (PI3K)/AKT, MAPK and SRC pathways in the cell lines with acquired resistance to BRAF inhibition. In cells with intrinsic resistance, we found that EGFR, MET and pAKT were relatively higher than in sensitive cells, while SRC phosphorylation was absent.

Hence, these data further support that, first, altered expression of selected kinases is reflected in kinome profiling, second, acquired resistance occurs via a common mechanism of increased kinase activity.

**Figure 4:**
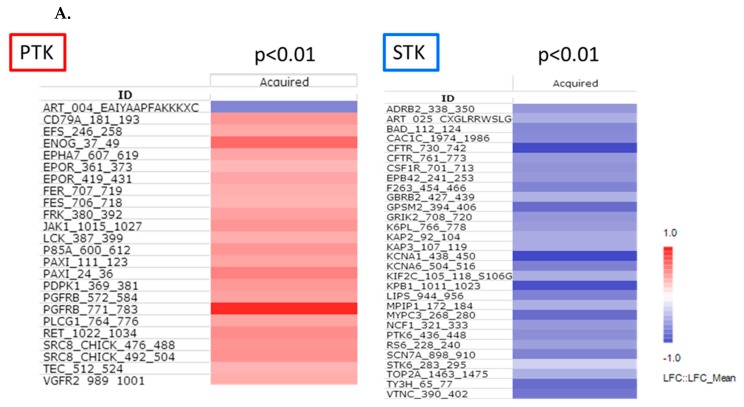

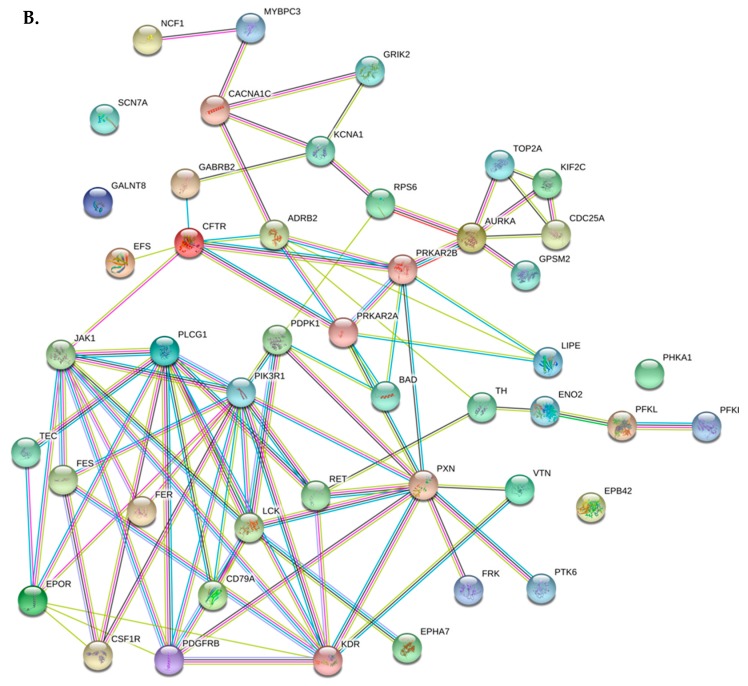
Common effects in cells lines with acquired resistance. (**A**) Common effects in the four cell line pairs determined with mixed model analysis. Peptides that were significantly differently (*p* < 0.01) phosphorylated in sensitive and resistant cell line pairs (^2^log fold change vs. sensitive cell lines). Red—increase, blue—decrease in resistant cell line; (**B**) STRING network analysis of all proteins with *p* < 0.01 in PTK or STK analysis.

To identify common effects of acquired resistance on kinase activity, a linear mixed model (ANOVA-like statistical model that contains both fixed (resistance) and random (cell line) factors) was used to identify the effects that are common to the four cell lines (Figure 4A). For visualisation, the differences in the kinome profile for BRAFi resistant cells relative to their parental cell line are shown as log ratios.

The combined PTK and STK data sets were used for a network analysis. Uniprot IDs of the peptides that were differentially phosphorylated with *p* < 0.01 were used for a STRING network analysis (Figure 4B). Each protein is represented as a node with edged interactions. This network analysis revealed that proteins with a strong functional interactive pattern include receptor tyrosine kinases (e.g., EGF-R, EPO-R, PDGF-R, VEGF-R2 (aka KDR)) and their downstream cytoplasmic kinases (e.g., SRC, JAK (Janus kinase), FER (Feline encephalitis virus-related kinase), FES (Feline sarcoma/Fujinami avian sarcoma oncogene homolog), Ret (Rearranged during transfection), TEC (Tyrosine-protein kinase Tec), LCK (Lymphocyte-specific protein tyrosine kinase)) that culminate in phospholipase C gamma (PLCgamma) and PI3K-mediated signalling that drives STK-mediated (PKA (i.e., PRKAR2A and PRKA2B), PDK1 (3-phosphoinositide-dependent protein kinase 1, a.k.a. PDPK1)) changes in ion channels (e.g., ADBR2, CAC1C, CFTR, KCNA1, KCNA6, SCN7A, GALNTB), cell cycle related proteins (e.g., AurA, TOP2A, KIF2C, CDC25A, GPSM2) and regulators of metabolism (TH, ENO2, LIPE, PFKL, PFK, PHKA1).

Compared to the findings for patient samples, the focus shifted to receptor and cytoplasmic tyrosine kinases (bottom part of the network). The contribution of MEK, AKT and mTOR to acquired resistance is very cell line-dependent, although a strong link to STK activity via PI3K and PDK1 is suggested. Most of the kinases cited above with a significant change in activity between sensitive and resistant cells are also involved in the MAPK, PI3K and SRC networks and regulate cell survival and cell migration.

### 3.4. BRAFi and MEKi Combination Effect on STK and PTK Activities in Cell Lines 

**Figure 5:**
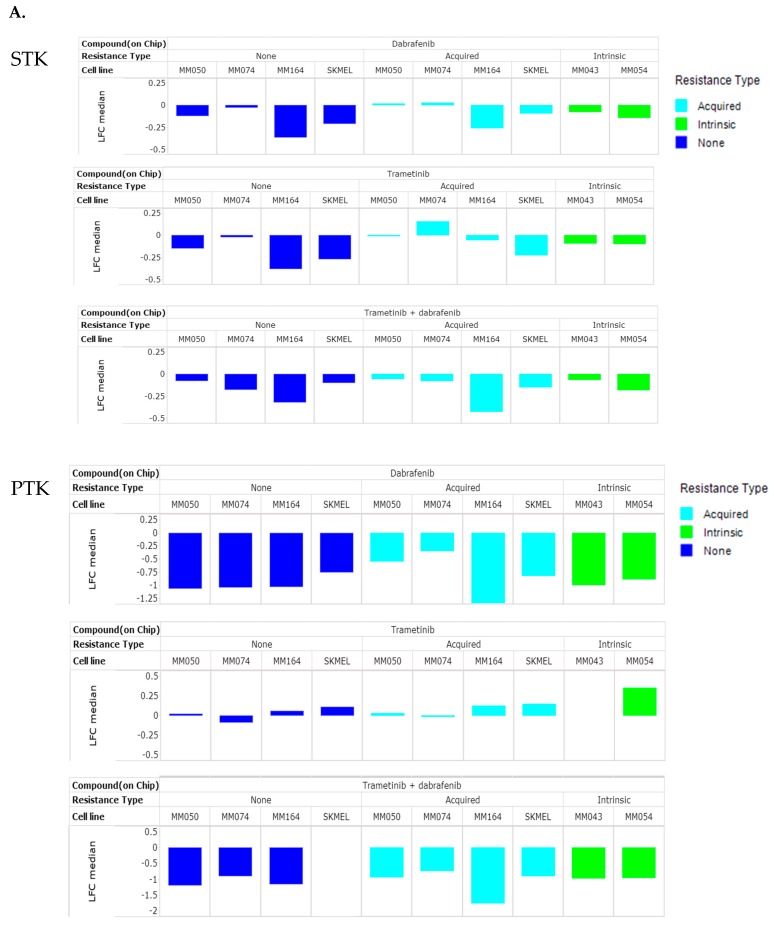

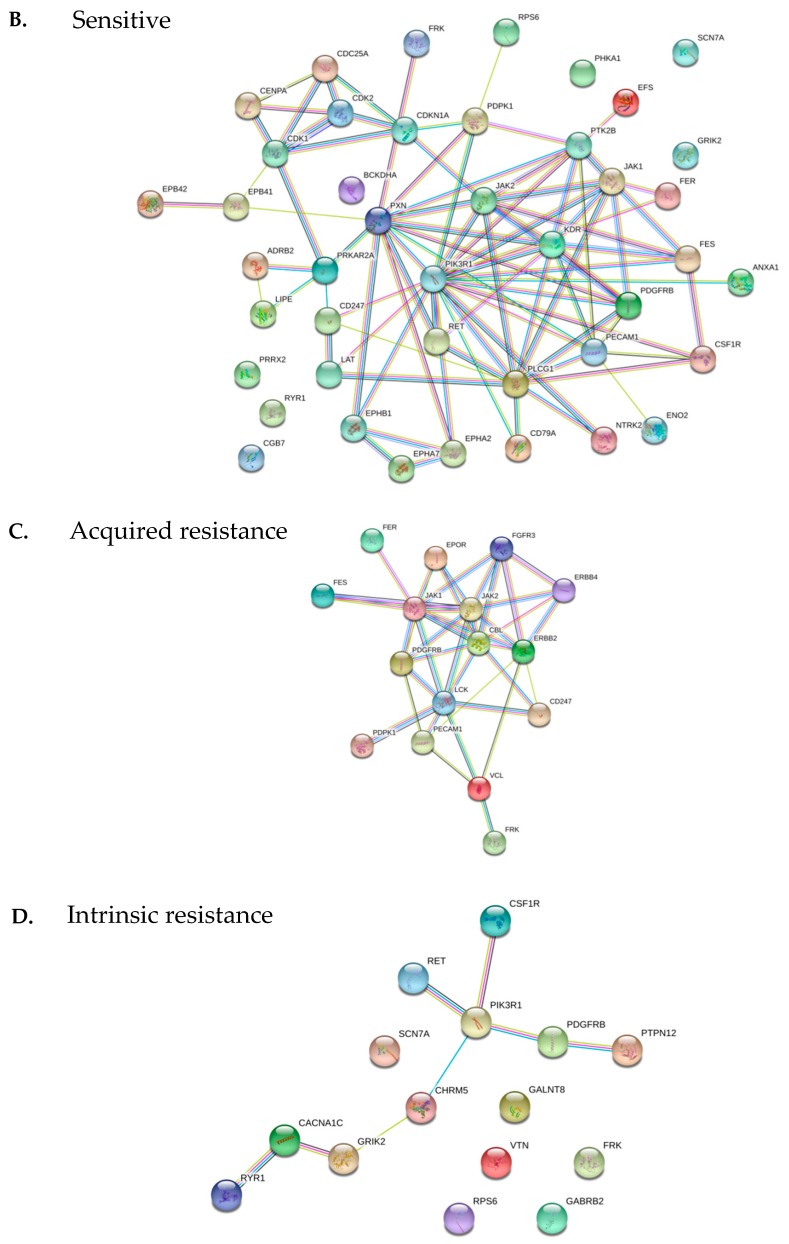
Effect of dabrafenib, trametinib and their combination on STK and PTK kinase activities. (**A**) Median Log Fold Change of all peptides (median of LFC for all peptides of replicate experiments) of serine/threonine or tyrosine kinase activity upon addition of dabrafenib, trametinib or the combination to the kinase assay. Cell lines are coloured by resistance type; (**B–D**) Network representation (STRING) of peptides that show a significant interaction between dabrafenib and trametinib in sensitive cell lines (**B**), cell lines with intrinsic (**C**) or acquired resistance (**D**).

Our next aim was to evaluate the effect of dabrafenib and trametinib, alone or in combination, on the kinase activity of cell lines with acquired or intrinsic resistance to vemurafenib in comparison with parental cells. Here too, the inhibitor(s) was added to the lysates just prior to the assay. We reported that dabrafenib is a more potent inhibitor of PTK activity than of STK activity, both in the cell line and in the melanoma tissue lysates. Here, we observed that 10 µM trametinib resulted in overall inhibition of STK activity, whereas a slight activation was observed on PTK (Figure 5A). Treatment with the combination resulted in inhibition in both assay types.

The interaction between dabrafenib and trametinib was investigated in the resistant versus sensitive cell pair using a two-way ANOVA mixed model analysis to identify common effects. On PTK 18 and 33 peptides were significantly (*p* < 0.05) associated with the combined treatment for the acquired resistant and sensitive cell lines, respectively (eight peptides in common), and five for the intrinsic resistant cell lines. On STK, no interaction between dabrafenib and trametinib was found for the cell lines with acquired resistance, but there was interaction for the sensitive and intrinsic cell lines (11 peptides in common (*p* < 0.05) for intrinsic and 16 peptides for the sensitive cell lines). The network representations of these peptide sets show that many nodes are affected by the combination of dabrafenib and trametinib in the sensitive cell lines (Figure 5B). The cell lines with acquired resistance show the effect of the combination on tyrosine kinases, whereas the intrinsic resistant cell lines have a completely different pattern.

When the interaction between dabrafenib and trametinib was investigated per cell line, a strong interaction was found for SKMEL-28 on both PTK and STK activity. MM164 showed interaction between the compounds on STK, and MM164-R on PTK. For the other cell lines, interaction was found on only a few peptides, which differed per cell line.

## 4. Discussion

In many cancers, deregulated kinase activities, often caused by activating mutations, result in hyperactivity, leading to uncontrolled cell growth [36]. Treatment of tumours with targeted kinase inhibitors has been shown to be initially very effective, but often resistance emerged. In this context, unprecedented responses have been observed in BRAF mutated melanoma after treatment with BRAF V600E targeted therapy [15]. Unfortunately, resistance developed in all patients [22]. A global analysis of all kinase activities (the kinome) can be very helpful both to predict response to MAPK inhibitors before treatment and to understand such mechanisms of resistance.

Indeed, a study using kinase activity profiling showed that the ex vivo effect of the multikinase inhibitor sunitinib in renal cell carcinoma (RCC) is positively correlated with clinical response [37]. Phosphorylation profiling as potential biomarker to predict tumour response to targeted therapy (kinase inhibitors) has been also investigated recently in RCC, as well (NCT02071719), further supporting the use of kinome profiles as predictive biomarkers. In our study, we compared kinase activities in tumour biopsies of responder and non-responder-patients and used cell lines that are sensitive or with innate/acquired resistance to MAPK inhibition in order to possibly propose kinome signatures of resistance to BRAFi.

Biological and technological advances have been developed to identify new targets for drug development. RNA interference (RNAi) [38], genomics [39], and proteomics [40] based methods have shown promise in these types of studies. However, these assays are limited in their ability to analyse the kinase pathways and the interplay between kinases. The assessment of protein phosphorylation status in response to therapeutic interventions is a challenge, due to the transient and complex nature of the phenomenon [41]. To date, the number of phosphorylation events that can be studied at one time is limited [41]. Thus, kinome analysis of biological systems provides a more direct measure of kinase signalling networks by determining changes in kinase activity (as phosphorylation events). In this study, we used a three-dimensional peptide chip microarray (PamChip® microarray), a platform that measures 284 serine/threonine (STK) and tyrosine (PTK) phosphorylation events with a kinase assay, in material obtained from fresh-frozen tumour samples and cell line lysates. 

We evaluated the inhibition profiles obtained after ex vivo exposure of tumour tissue lysates to MAPK inhibitors and found that phosphorylation levels of several kinase substrates differed between patients who were responding and not responding to BRAF inhibition monotherapy. Inhibition of STK activity in sensitive samples by dabrafenib and trametinib in combination was stronger, indicating a high dependency on the BRAF pathway in these responder patients (Figure 2). However, at basal level, due to the biological variation in the limited number of samples, only a few peptides were differentially phosphorylated between responders and non-responders. Conversely, ex vivo treatment with a ^V600E^BRAF inhibitor could identify robust common features that discriminate responders from non-responders. Furthermore, both in melanoma tissues and cell lines, the BRAF inhibitor was found to be more potent as a PTK inhibitor than as a STK inhibitor, in accordance with Tahiri et al, who found higher inhibition of PTK activity by vemurafenib in ^V600E^BRAF melanoma than in BRAF WT [42], indicating that ^V600E^BRAF mainly activates signalling pathways implicating tyrosine kinases.

Interestingly, kinases within the MAPK, PI3K/AKT and SRC signalling networks (summarised in Table S3) that are involved in cancer progression and resistance to therapy were among those discriminating responders from non-responders, indicating that these pathways, leading to cancer cell proliferation and survival, are more active in melanoma of responding patients. Moreover, we performed comparative analysis of kinome profiles under MAPK inhibition among melanoma cell lines that are sensitive to the ^V600E^BRAF inhibitor vemurafenib or with intrinsic or acquired resistance to the drug, and we showed significant differences in phosphorylation profiles between the two groups (sensitive vs. resistant). 

To further understand and gain more insight into the mechanisms of acquired resistance promoted by adaptive changes of the kinome profile in response to MAPK inhibitors, we performed two types of analyses: a protein–protein interaction network and an identification of direct upstream kinases. The most relevant kinases with strong interactive patterns present within the network include tyrosine kinase receptors (EGFR, Platelet derived growth factor receptor (PDGFR)α/β, insulin-like growth factor 1 receptor (IGFR1R) and RET), AKT and SRC.

Furthermore, PDPK1 and PIK3R1 (p85α regulatory subunit of PI3K), two main components of the PI3K/AKT pathway, were also present in the identified protein pattern. Several studies have reported a central role for the PI3K/AKT pathway in the resistance to ^V600E^BRAF inhibition therapy [23,43,44]. JAK1 and SRC are also important nodes found in the interaction analysis. In addition to SRC, other SRC family members such as FRK (Fyn related kinase), LCK, HCK (hematopoietic cell kinase), FYN (Proto-oncogene c-Fyn), YES (Yamaguchi sarcoma oncogene YES) and LYN (Yamaguchi sarcoma viral related oncogene homolog Lyn) are also represented in the interaction network, underlining the strong implication of such kinases in the resistance mechanisms as multiple kinases also involved in the same pathways are identified from our analyses.

Moreover, EpoR (erythropoietin receptor), also displayed in the interaction network, can promote angiogenesis, melanoma cell survival and has been reported to counteract cisplatin-induced cell death [45,46]. Interestingly, the interaction network identified additional kinases that are involved in cancer progression but that have not been previously associated with melanoma, such as paxillin, PLCG1 and TEC (Figure 5B–D). Thus, on top of the numerous published studies [47] depicting mechanisms associated with acquired resistance to MAPK inhibition, our study points to other members of alternative pathways that should be validated and considered as new targets in combination treatment strategies.

In a next step, kinase profiling was validated by Western blot. The results revealed an upregulation of RTK (EGFR and c-MET) and an activation of MAPK, PI3K/AKT and SRC signalling networks, confirming our findings in patients as well as in cell lines (Figure 3C). Indeed, the cell lines with either intrinsic or acquired resistance showed an increase in PKA and AKT/mTOR activities through the activation of RTK, SRC and PI3K. However, we found in a previous study that the resistance to vemurafenib is effectively due to an activated PI3K/AKT pathway in MM043 cells, and PI3K inhibitors tested in this study confirm the role of AKT in overcoming BRAFi on BRAF^V600E^ cells [23].

Thus our results clearly support the involvement of PI3K/AKT pathway activation in vitro and in vivo by various converging mechanisms.

Furthermore, this technology offered us the opportunity to compare sensitive and resistant cell lines to BRAFi with the aim to dissect the global impact of adding MEKi on kinase activity. In sensitive cells, BRAFi induced a stronger kinase inhibition than the one obtained with MEKi alone. This could be in line with the finding that MEKi monotherapy is less effective than BRAFi monotherapy in terms of response rate [48]. In resistant cells, the picture is different, favouring a much higher effect of BRAF/MEK inhibitor combination in accordance with clinical data that led to the latter combination becoming the standard of care.

Lastly, cAMP/PKA are known to have a particular role in overcoming resistance to BRAF V600E inhibitors [26,49,50], e.g., PKA is able to inactivate the CRAF by phosphorylating serines S43, S233 and S259 [51,52]. The same applies for melanoma cells with BRAF and NRAS wild-type proteins as we demonstrated in a previous study [26]. Of note, all sensitive cell lines used in the present study have significantly higher cAMP levels and consequently higher PKA activity than the resistant ones, resulting in a more potent inhibition of the CRAF activity, thus contributing to the efficacy of BRAFi in such cells by limiting the activation of an important resistance pathway [26].

## 5. Conclusions

In conclusion and despite the limited number of patient biopsies, our study proposes a kinome profile using a reliable multiplex assay platform able to predict treatment outcome. Our findings will need a larger validation cohort in a prospective study to draw definitive conclusions. Nonetheless, one of the tumour tissue biopsies that we used in this study (518) originates from a patient under vemurafenib monotherapy without evidence of any recurrences for more than five years now, showing the long-term efficacy of such treatment in certain patients. Interestingly, our study showed that the following kinome profile, together with a relatively high cAMP/PKA pathway activity may explain this long-lasting tumour control.

## Supplementary Materials

The following are available online at https://www.mdpi.com/2072-6694/12/2/512/s1, Figure S1: Kinase activity profiling on PamChip^®^ peptide microarrays (**A**) Phosphorylation of peptides on chip is monitored (**B**, **C**). Control measurement (**C1**) and ex vivo compound addition (**C2**). Signal intensities are converted to kinase activity profiles (**D**) and to inhibition profiles (**E**). Figure S2: A. Four melanoma cell lines (MM050, MM074, MM164 and SKMEL-28), all with BRAF V600E mutations, were made resistant to the BRAF V600E inhibitor vemurafenib after exposure during 12 weeks to gradually increasing concentrations of vemurafenib (0.1–2 µM). B. Growth inhibition IC50 values after three days of vemurafenib exposure in parental and resistant cell lines. C. Effect of increasing concentrations (10^−8^–10^−4^ M) of vemurafenib for three days (crystal violet assay). Proliferation is expressed as means ± SD (*n* = 3) compared to untreated cells (CTR). Figure S3: Upstream kinase analysis of changes in kinase activity in resistant cell line pairs. Green—increased activity in resistant cell lines, red—lower in resistant cell lines. Illustration reproduced courtesy of Cell Signaling Technology, Inc. (www.cellsignal.com) using Kinmap. Table S1: Description of BRAF mutant melanoma cell lines by melanoma type, metastasis site of which they were derived, BRAF, NRAS and PTEN mutation status and sensitivity to vemurafenib (growth inhibition IC50 values). Table S2: Characteristics of melanoma samples and patients. All tumours express ^V600E^BRAF and all patients were treated with vemurafenib. Table S3: Peptides that show significant difference in inhibition between responder and non-responders melanoma patient tissues, Peptide IDs, UniProt IDs and STRING names. Table S4: Peptides that show significant difference between cell lines with acquired resistance vs. parental cells, Peptide IDs, UniProt IDs and STRING names.
