# Erratum: Krayem, M., et al. Kinome Profiling to Predict Sensitivity to MAPK Inhibition in Melanoma and to Provide New Insights into Intrinsic and Acquired Mechanism of Resistance Short Title: Sensitivity Prediction to MAPK Inhibitors in Melanoma. *Cancers* 2020, *12*, 512

**DOI:** 10.3390/cancers12102981

**Published:** 2020-10-14

**Authors:** Mohammad Krayem, Philippe Aftimos, Ahmad Najem, Tim van den Hooven, Adriënne van den Berg, Liesbeth Hovestad-Bijl, Rik de Wijn, Riet Hilhorst, Rob Ruijtenbeek, Malak Sabbah, Joseph Kerger, Ahmad Awada, Fabrice Journe, Ghanem E. Ghanem

**Affiliations:** 1 Laboratory of Oncology and Experimental Surgery, Institut Jules Bordet, Université Libre de Bruxelles, 1000 Brussels, Belgium; ahmad.najem@bordet.be (A.N.); msabbah@ulb.ac.be (M.S.); ahmad.awada@bordet.be (A.A.); fabrice.journe@bordet.be (F.J.); gghanem@ulb.ac.be (G.E.G.); 2 Medical Oncology Clinic, Institut Jules Bordet, Université Libre de Bruxelles, 1000 Brussels, Belgium; philippe.aftimos@bordet.be (P.A.); joseph.kerger@bordet.be (J.K.); 3 PamGene International BV, 5211HH ‘s-Hertogenbosch, The Netherlands; timvdhooven@gmail.com (T.v.d.H.); avdberg@pamgene.com (A.v.d.B.); lhovestad@pamgene.com (L.H.-B.); rdwijn@pamgene.com (R.d.W.); rhilhorst@pamgene.com (R.H.); rru@genmab.com (R.R.)

The authors would like to make a correction to their published paper [1].

The authors would like to change the title from “Kinome Profiling to Predict Sensitivity to MAPK Inhibition in Melanoma and to Provide New Insights into Intrinsic and Acquired Mechanism of Resistance Short Title: Sensitivity Prediction to MAPK Inhibitors in Melanoma” to “Kinome Profiling to Predict Sensitivity to MAPK Inhibition in Melanoma and to Provide New Insights into Intrinsic and Acquired Mechanism of Resistance”. 

We stress that this correction does not change the written portion of the figure legend, interpretation of results, or final conclusion of this manuscript. The authors would like to apologize for any inconvenience caused. The original article has been updated.
