# Correction: Krayem et al. Kinome Profiling to Predict Sensitivity to MAPK Inhibition in Melanoma and to Provide New Insights into Intrinsic and Acquired Mechanism of Resistance. *Cancers* 2020, *12*, 512

**DOI:** 10.3390/cancers16142587

**Published:** 2024-07-19

**Authors:** Mohammad Krayem, Philippe Aftimos, Ahmad Najem, Tim van den Hooven, Adriënne van den Berg, Liesbeth Hovestad-Bijl, Rik de Wijn, Riet Hilhorst, Rob Ruijtenbeek, Malak Sabbah, Joseph Kerger, Ahmad Awada, Fabrice Journe, Ghanem E. Ghanem

**Affiliations:** 1 Laboratory of Oncology and Experimental Surgery, Institut Jules Bordet, Université Libre de Bruxelles, 1000 Brussels, Belgium; ahmad.najem@bordet.be (A.N.); msabbah@ulb.ac.be (M.S.); ahmad.awada@bordet.be (A.A.); fabrice.journe@bordet.be (F.J.); gghanem@ulb.ac.be (G.E.G.); 2 Medical Oncology Clinic, Institut Jules Bordet, Université Libre de Bruxelles, 1000 Brussels, Belgium; philippe.aftimos@bordet.be (P.A.); joseph.kerger@bordet.be (J.K.); 3 PamGene International BV, 5211HH ’s-Hertogenbosch, The Netherlands; timvdhooven@gmail.com (T.v.d.H.); avdberg@pamgene.com (A.v.d.B.); lhovestad@pamgene.com (L.H.-B.); rdwijn@pamgene.com (R.d.W.); rhilhorst@pamgene.com (R.H.); rru@genmab.com (R.R.)

## Error in Figure

In the original article [1], there was a mistake in Figure 3C as published. The ERK, AKT, and SRC blots in our publication in *Cancers* (Figure 3C) were flipped when resizing the images. Note that this mistake does not change or alter the content and conclusions of our paper. The mistake indeed concerns control experiments, which were reproduced very easily. The corrected Erratum Figure 3C appears below.

The pERK that appears on the third line for the conditions MM074 and MM074-R is correct, and the error came from another paper published in *Oncotarget* (https://doi.org/10.18632/oncotarget.25879) that was retracted.

In the original article, the first author's name is missing an m, and the correct version is Mohammad Krayem.

The authors apologize for any inconvenience caused and state that the scientific conclusions are unaffected. This correction was approved by the Academic Editor. The original publication has also been updated.

## Figures and Tables

**Figure 3:**
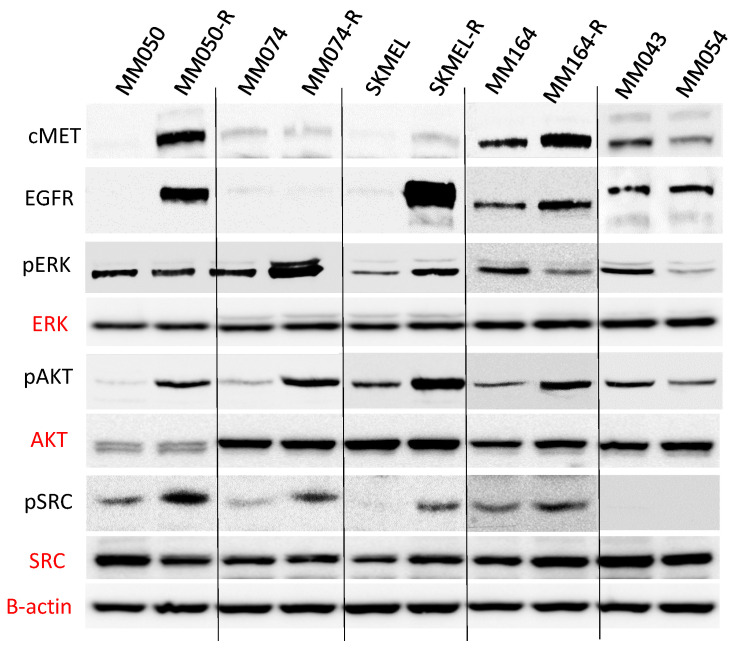
(**C**) Western Blot analysis of key targeted protein in MAPK, PI3K/AKT and SRC signalling pathways in four BRAFi sensitive, and six cell lines with intrinsic (MM043 and MM054) and acquired resistance to vemurafenib, (R refers to acquired resistant cells compared to parental sensitive cells).
