# Supplementary material for: Kinome Profiling to Predict Sensitivity to MAPK Inhibition in Melanoma and to Provide New Insights into Intrinsic and Acquired Mechanism of Resistance : Short Title: Sensitivity Prediction to MAPK Inhibitors in Melanoma
Source: Cancers (Basel). 2020 Feb 22;12(2):512. doi: 10.3390/cancers12020512 (PMC7072684; doi:10.3390/cancers12020512)
Supplement: Supplementary file 1 [file cancers-12-00512-s001.zip › Table S1.docx]

**Table 1.** Description of BRAF mutant melanoma cell lines by melanoma type, metastasis site of which they were derived, BRAF, NRAS and PTEN mutation status and sensitivity to vemurafenib (growth inhibition IC50 values).

| **Cell line** | **Melanoma type** | **Metastatic site** | **Resistance** | **Vemu IC50 (μM)** | **BRAF status** | **NRAS status** | **PTEN status** |
| --- | --- | --- | --- | --- | --- | --- | --- |
| MM050 | SSM | LN | NA | 0.1 | V600E | WT | WT |
| MM050-R | SSM | LN | Acquired | 30 | V600E | WT | WT |
| MM074 | SSM | LN | NA | 0.1 | V600E | WT | WT |
| MM074-R | SSM | LN | Acquired | 20 | V600E | WT | WT |
| SKMEL | SSM | LN | NA | 0.3 | V600E | WT | WT |
| SKMEL-R | SSM | LN | Acquired | 20 | V600E | WT | WT |
| MM043 | SSM | Intestine | Intrinsic | 20 | V600E | WT | WT |
| MM054 | NM | SK | Intrinsic | 40 | V600E | WT | WT |
