# Supplementary material for: Kinome Profiling to Predict Sensitivity to MAPK Inhibition in Melanoma and to Provide New Insights into Intrinsic and Acquired Mechanism of Resistance : Short Title: Sensitivity Prediction to MAPK Inhibitors in Melanoma
Source: Cancers (Basel). 2020 Feb 22;12(2):512. doi: 10.3390/cancers12020512 (PMC7072684; doi:10.3390/cancers12020512)
Supplement: Supplementary file 1 [file cancers-12-00512-s001.zip › Table S2 - Patients.docx]

**Table S2 :** Characteristics of melanoma samples and patients. All tumors express V600E BRAF and all patients were treated with vemurafenib.

| **Samples** | **Melanoma type** ^a^ | **Metastatic site** ^b^ | **Response to vemurafenib** | **Duration of vemurafenib treatment (months)** | **OS**  ^c^ **(months)** | **OS status** |
| --- | --- | --- | --- | --- | --- | --- |
| 467 | NM | LN | Responder | 4 | 65 | dead |
| 499 | SSM | LN | Responder | 9 | 70 | dead |
| 516 | SSM | LN | Responder | 39 | 101 | dead |
| 518 | Unknown | LN | Responder | 89 | 92 | alive |
| 524 | Unknown | LN | Non responder | 4 | 23 | dead |
| 525 | NM | SK | Non responder | 3 | 79 | dead |
| 549 | SSM | SK | Non responder | 4 | 28 | dead |

^a^ NM: nodular melanoma, SSM: superficial spreading melanoma

^b^ LN: lymph node metastasis, SK: skin metastasis

^c^ OS: overall survival
